# Teacher Authority in Long-Lasting Cases of Bullying: A Qualitative Study from Norway and Ireland

**DOI:** 10.3390/ijerph16071163

**Published:** 2019-03-31

**Authors:** Ida Risanger Sjursø, Hildegunn Fandrem, James O’Higgins Norman, Erling Roland

**Affiliations:** 1 Norwegian Centre for Learning Environment and Behavioral Research in Education, University of Stavanger, 4036 Stavanger, Norway; hildegunn.fandrem@uis.no (H.F.); erling.roland@uis.no (E.R.); 2 The National Anti-Bullying Research and Resource Centre, Dublin City University, St. Patrick’s Campus, Dublin 9, Ireland; james.ohigginsnorman@dcu.ie

**Keywords:** traditional victimization, cyber victimization, bullying, teacher styles, authoritative leadership, warmth, control, class teacher

## Abstract

A growing body of research shows a correlation between an authoritative school climate and lower levels of bullying. One objective of this study is to conceptualize authoritative intervention in bullying cases. A second goal is to explore whether, and how, the pupils, having experienced traditional and/or cyber victimization, perceive that the class teacher is demonstrating authoritative leadership when intervening in long-lasting cases of bullying. Class teacher refers to the teacher that has a special responsibility for the class. The article presents the findings from nine semi-structured interviews with four Irish and five Norwegian pupils. The informants were between 12 to 18 years of age and had experienced either traditional victimization or both traditional and cyber victimization for 1 to 7 years. The informants were selected because their cases had been reported as resolved. The findings showed no descriptions of the class teacher that appeared to fit with the authoritative style of leadership, both high on warmth and control. The possible practical implications of these findings are discussed.

## 1. Introduction

Bullying can change a victim’s view of the people surrounding them. It can lead to lack of trust and disappointment towards peers and a lack of involvement, but it can also evolve into disappointment in teachers. This highlights the importance of having caring and competent teachers in school who make it easier for the pupils to cope with bullying.

### 1.1. Authoritative Classroom Leadership

The authoritative style, which is high on control and high on warmth, appears to be the adult role that produces the best results for a child’s development [1,2,3]. However, this important insight does not necessarily mean that such leadership is the best way to handle bullying.

When studying the concept of authoritative classroom leadership [3,4,5,6], research refers to the first of four parental styles, introduced by Baumrind [1,2]. The four styles are based on two dimensions: Degree of control/demands and degree of warmth/nurturance. Authoritative (high on warmth, high on control), authoritarian (low on warmth, high on control), indulgent (high on warmth, low on control), and neglectful (low on warmth, low on control) are the four styles [1,2]. Control could be defined as “enforcing demands for appropriate behavior” [3] (p. 123). Warmth could be defined as supporting the child’s agency and individuality in addition to being sensitive and responding to the needs of the child [3].

Roland and Galloway [7] found a positive correlation between teacher authority in the classroom and low levels of bullying and that improved teacher authority in the classroom reduced bullying [8]. One study has found a relationship between victimization and classroom climate that has low levels of caring, warmth, and support [9]. Thornberg, Wänström, and Jungert [10] found that students belonging to a classroom with an authoritative climate, measured from the students’ perspective, were less likely to experience victimization in school. In addition, they found that an authoritative classroom appears to be related to greater defense and less reinforcement from peers when bullying is happening.

Bullying is predominantly proactive aggression [11,12] and adult control reduces this aggression [7]. Teacher authority also tends to improve teacher–pupil relations and thereby pupil–pupil relations, which stimulates the pupils to support and protect each other [7]. This could indicate that authoritative leadership on the part of the teacher is positive when intervening with bullying, and not just for prevention. To our knowledge, there have been no studies looking directly at teacher style in relation to intervening for victims.

### 1.2. Perceived Authoritative Intervention

A challenge is that authoritative classroom leadership is described as how to address one unit, for example a pupil or a class [3,7]. A bullying case is a strongly differentiated social system comprising at least a bully and a victim. Furthermore, according to Marzano [13], different reactions to, or aspects of, the authoritative style is important to emphasize dependent on what behavior is shown. Thus, teacher authority may be differently perceived according to the roles one has in such a case.

Victim-perceived authoritative intervention has, therefore, to be defined according to teachers’ warmth and control towards both the victim and one or more bullies, as the victim presumably sees it. It is reasonable, to assume that the victim wants the teacher to demonstrate warmth towards her or himself, in other words that the warmth dimension of authority is highly relevant for the victim. Another question is how the teacher should calibrate such empathy towards a child who is suffering from bullying. What the victims say about warmth related concerns they receive from the teacher is important.

The control dimension of authority is different from warmth when seen from the victims’ perspective. When the communication with the teacher is about the ongoing bullying, the victims may feel humiliated if the teacher profiles control towards them. The control dimension is, however, interesting when related to the safety of the victim, and we suggest calling this ‘perceived protective control’. By this, we mean whether the victim realizes that the teacher is willing to stop the bullying and/or capable of stopping the bullying. Again, it is interesting to disclose how the victims of long-lasting bullying discuss this.

### 1.3. Aims of the Study

We explored how the victims described the bullying situation, and how the cases ended. The first objective was to conceptualize authoritative intervention in bullying cases, from the assumed perspective of the victim. A second goal was to explore the experiences and understanding of pupils who have been bullied in regard to how teachers responded when the bullying was occurring.

## 2. Method

### 2.1. Sample

A convenience sample was used [14]. We used this because of the challenge of finding informants who have been victims of bullying and who were willing to be interviewed. In our case, professional connections were used, and the sample consisted of cases reported to the national centers working with bullying in Norway and Ireland. The inclusion criteria for the sample were that the cases should have been considered as bullying according to a standard definition: A negative repeated act, against someone who cannot easily defend themselves [11,12,15] resolved within the last year, the pupils had not themselves bullied others and the victim was comfortable talking about the victimization. In addition, to increase the possibility of finding informants, the age span was set from 8 to 18 years.

The informants were interviewed in Norway and Ireland in 2014. The school administration had reported the cases as resolved in Norway, and the Irish cases were resolved cases reported to the center. We interviewed 10 informants aged 8 to 18 years using semi-structured interviews: Of the 10 informants, 6 were from Norway and 4 from Ireland, including 2 boys and 8 girls. One interview provided too little information about the teacher and was therefore excluded. In all, there were 9 cases: 2 cases were victims who had experienced traditional bullying and 7 cases were victims who had experienced both traditional and cyberbullying. No cases included only cyberbullying.

### 2.2. Access and Ethics

The ethics committees in both Ireland and Norway received information about the study and both committees approved the study. Prior to the study, the informants and the parents received letters with information about the study. The parents with children under the age of 18 were asked to fill out a parental consent form before they were contacted by phone to set a date for the interview. All the informants below the age of 18 were given information about the study customized to their age. They were also told that they could withdraw from the study at any time. Being a member of a vulnerable group also gives these children an important voice and could, as Dalen [16] asserts, contribute to new and important information that could improve the situation for children experiencing similar events. 

### 2.3. Data Collection

The data collection was conducted using individual qualitative semi-structured interviews, jointly constructed by the members of the research team. Originally, the interview guide was written in Norwegian and translated into English for the interviews in Ireland. Members of the research group addressing bullying were the ones conducting the interviews. In Ireland, a Norwegian, who is also fluent in English, conducted all interviews to secure the same meaning in the two countries. The interview guide had different themes that were to be explored with the participants. The main themes were: Their experience of being bullied from the beginning of the bullying to the resolution of the situation, some of the episodes they remembered well, communication about the bullying, if and how the bullying affected their life in general, the investigation and what was done by the school, what measures were attempted and what social support did they receive, how they felt emotionally and how the bullying affected their life in school and also their spare time, their relationships with the teacher, parents, and other pupils.

The interviews were held in a private setting or a private room in the school, lasted from 30 to 90 min, and were tape-recorded. The interviews started with small talk, to make the informants as relaxed as possible before they were asked to talk about their bullying experience. The same definition of bullying was presented to all the informants before starting the interview. The definition stated that bullying is a repeated aggressive act, including an imbalance of power, against someone who cannot easily defend themselves. The interviews were transcribed using standardized methods agreed upon in the research group, inspired by the description given in Kvale and Brinkmann [17].

### 2.4. Data Analysis

The first step in the analysis of the interviews was to read through of all the interviews to get an overview of emerging themes regarding how teachers were described. The interviews where read through a second time, and during this read through all the descriptions of the teachers were gathered under one main node ‘teacher descriptions’. In further analysis, everything that was gathered under ‘teacher descriptions’ was read through and only the descriptions related to the class teacher were chosen for a new main node ‘class teacher’. As we read through the node ‘class teacher’ focusing on themes relating to the class teachers support of the informants, two themes emerged; the class teacher offering emotional support, and the class teacher stopping the bullying. The second step was to use a thematic approach to identify, analyze, and report on patterns or themes that were found within the data [18]. Rather than an inductive, a theoretical approach was used, fitting the data into pre-existing coding frames [17] i.e., the two aspects of the authoritative teacher style; warmth and control. In this step of the analysis, also a semantic rather than a latent approach was used, meaning that what a participant said was more important than going beyond the semantic content of the data to identify the underlying ideas, assumptions, and conceptualizations [18]. For analysis, the qualitative data analysis software NVivo 12 (QSR International, Melbourne, Australia) was used. This program was used for storage and sorting of the data in addition to being helpful in the process of categorizing and classifying the data thematically. The first read through was done by two researchers, while the latter analysis was done by only one researcher, but with a continuous check of the interpretations by one or two other researchers in the team. If there were disagreements, a third party was involved.

## 3. Findings

The first section will cover descriptions of how the bullying happened and how it stopped. Further, the findings will be presented relating to the two main themes that occurred from our data: Class teacher showing control and class teacher showing warmth. As different degrees and aspects of warmth and control shown by the class teacher were described, the main node of control and warmth was divided in to lacking, low, medium, and high. A teacher that had a high or medium on both control and warmth would be categorized as authoritative. The findings, however, showed that none of the nine informants perceived their class teacher as having had a high or medium score on both warmth and control. The findings regarding the main nodes of control and warmth will, therefore, be presented separately. Later in the process, we changed control to ‘protective control’.

### 3.1. How Did the Bullying Happen and How Did It Stop

The informants were all asked how the bullying happened. None of them described having experienced physical bullying. The most common descriptions were negative comments and exclusion, both in real life and online. For the victims that had experienced both types of victimization, the bullying was often described as starting with exclusion and negative comments in real life, which then followed to similar occurrences online for example on the social media platform, Instagram. Only one of the informants, having experienced both traditional and cyber victimization, described being victimized by someone else online than the ones they were interacting with in the school setting.

When describing how the bullying stopped, none of the informants described their class teacher being directly involved in this. Over half of the informants changed school, describing this as a new start. A couple of the informants experienced that the bullying just stopped without any special measures being done. The last informant isolated herself from the other pupils and explained that this was the reason that the bullying stopped.

### 3.2. Class Teachers and Perceived Protective Control

The content of all the descriptions related to the perception of the class teacher as lacking or low on protective control impacted on the victim’s perceived belief that the teacher could stop the bullying. The class teachers were mainly perceived as passive or having low competence. On the question of what the teacher would do to stop the bullying, over half of the participants described their class teachers as lacking protective control: “They did not react to it” (Norwegian girl, 17), “The teachers were just completely ignoring that it happened, I didn’t get anything sorted” (Irish girl, 17). In addition to experiencing that the teachers did not react to their victimization with constructive measures, also a lack of communication about what the teacher thought and would do was described: “The teachers kept it to themselves” (Irish girl, 18), “The teacher actually did not say anything to me” (Irish girl, 13). A few of the class teachers were described as low on protective control. What classified the teacher being described as low on protective control were that they tried to intervene, but the informant described that the teacher had challenges due to possible lack of experience: “She tried everything she could, but it’s difficult when you don’t have the experience” (Norwegian girl, 14). “The teacher talked to us and it was very time consuming, I felt it only got worse, because then we could hear what the other one felt and we ended up getting mad at each other” (Norwegian girl, 13), “The teacher tried but he gave up because there was nothing to do” (Norwegian girl, 12). None of the class teachers were described as medium or high regarding protective control.

### 3.3. Class Teachers and Perceived Warmth

The perceptions of the degree of warmth shown by class teachers was revealed from the extent to which the informants reported they could talk to their teachers about bullying, as well as their general relationship with the class teacher. Warmth was also described as either lacking, low, medium, or high.

High on warmth is described as a good relationship where the informants experience that the teacher is there for them and that they can tell them everything and get the feeling of being heard. In our analysis, only one class teacher was found to be described in a way that could be interpreted as high on warmth: “She tried to help me, and she knew how I was feeling in a way. It was just like she was there for me, just like a friend kind of” (Norwegian girl, 14). Medium means they have an ok relationship and the informant and can tell the teacher about the experienced victimization. The informant trusts the teacher and tells him or her about the bullying. The teacher is described as an overall nice person to everyone, but not as having an especially good relationship with the informant. A couple of the informants described their class teacher as medium on warmth: “She was nice to everyone” (Norwegian girl, 13), “I don’t trust that many teachers really, but I trust my class tutor” (Norwegian girl, 12). A couple of informants also described their class teacher as low on warmth. With low, the informants described not talking much to their class teacher about having experienced the victimization and they described only telling their class teacher, but not having conversations about it: “I went to my tutor, my class tutor and I told her I was being bullied” (Irish girl, 18). Half of the informants described their class teacher in a way that could be related to a lack of warmth. To lack warmth refers to an absence of, or not a good, relationship described between the informant and their class teacher. They did not feel they could tell their class teacher what was going on. On the question if he could tell the teacher, one informant said “no” (Irish boy, 18). The informants also described it being difficult to trust their class teacher, “she pretends to be nice but she is not really” (Irish girl, 13). Another informant did not feel that she was taken seriously: “She meant I did not have a problem, so she did not listen to what I said. She only talked to me about my grades not to how I was doing” (Norwegian girl, 17). One informant described experiencing a situation where teacher supported the bullying taking place, “she encouraged the bullying. The teacher would never listen” (Irish girl, 17).

## 4. Discussion of Findings

One goal of the present study was to conceptualize teacher authority in responding to bullying, as perceived by the victim. We did this by relating the two main dimensions of authority—warmth and control [1,2] to the bullying core system, the victim and the bully or bullies, as perceived by the victim. From a theoretical perspective, we argued that a preferable teacher approach perceived by the victim would be warmth towards her or himself and control towards the bullies regarding their behavior. The other main goal, which was related to the first one, was to identify whether and how the class teachers demonstrate authoritative leadership in their work with long-lasting cases of bullying, from the perspective of the pupils having experienced victimization.

Our conceptualization of teacher authority related to a bullying case, as perceived by the victim, helped in analyzing the cases. The findings showed that none of these nine informants who experienced long-lasting cases of victimization gave a description of their class teacher that could be characterized as an authoritative teacher style regarding intervention to stop bullying. In other words, the nonappearance of teacher authority coincides with long-lasting bullying.

Our findings do not say that an authoritative style as conceptualized in this study would have hindered long-lasting bullying. This result is of interest and we will now focus on some of the possible reasons for the class teacher being perceived as low on control and/or low on warmth, or even lacking both aspects.

### 4.1. Lacking or Low on Control

The findings show that none of the participants perceived their class teachers as being medium or high on control. In fact, all participants perceived their class teachers as either lacking or low on control. There could be many reasons why a class teacher is perceived as low on control in a case of bullying. One reason could be the lack of competence in identifying bullying. Research has shown that teachers who experience bullying behavior as normative seem to be less likely to intervene and put an end to the bullying [19,20]. The teacher might not have understood that the child has been bullied and, therefore, did not find it necessary to act. Previous research has shown that teachers do not always know how to interpret what is happening, or do not know what bullying looks like [21]. Thus, a lack of competence could be related to uncertainty regarding what type of measures should be implemented. Research also has shown that when teachers believe that they lack the skills to be able to effectively intervene in a bullying situation, it decreases the possibility that they will intervene, and they are more likely to ignore the bullying [22]. One informant excuses her class teacher for not efficiently intervening in bullying as due to lack of competence: “She tried everything that she could, but it’s difficult when you don’t have the experience” (Norwegian girl, 14). When lacking competence, the teacher might decide to not do anything for fear of doing something that they think might be wrong. However, we argue that by not doing anything, the teachers are negative role models because they ignore bullying behavior and, in some cases, could actually be contributing to bullying children themselves [23]. One informant described her class teacher as one of the bullies: “She encouraged the bullying. The teacher would never listen so I gave up telling them” (Irish girl, 17).

### 4.2. Lacking or Low on Warmth

The findings show that only one participant perceived their class teachers as high on warmth, and only a couple described their class teacher as medium in this regard. Over half of the informants described their class teacher as low or lacking warmth. There could be several reasons why many of the class teachers seem to show little or no warmth towards the informants who experienced victimization. For instance, variations in teachers’ responses might reflect teachers’ lack of empathy [24]. Teachers who show empathy for others seem to be more likely to identify, report, and intervene when discovering bullying [22]. Some class teachers were described as not acknowledging the bullying and reacting with low empathy towards the child talking about their experience of victimization: “She meant I did not have a problem. So she did not listen to what I said” (Norwegian girl, 17). In relation to the empathy shown by the teacher, the type of bullying also seems to impact the quality of the teacher’s intervention. Teachers have less empathy and intervene less when witnessing relational, verbal, or cyber victimization compared to physical victimization [24,25]. Other studies showed that relational victimization is considered to be less serious than physical and verbal bullying [22]. However, the reasons for this difference are not necessarily a sign of a lack of empathy, but could be because physical bullying is easier to observe than relational and verbal victimization. None of the informants in this study described experiencing physical bullying, which might be easier to uncover. This lack of physical bullying might be one of the reasons why the cases lasted for such a long time.

## 5. Limitations of the Study

This study provides important qualitative information from informants who have experienced long-term victimization; however, there are some limitations. Finding children who had experienced victimization and agreed to be interviewed is not an easy task, and, therefore, a convenience sample was used.

The study only provides a retrospective perspective of the child, and the memories can be imprecise. This may particularly have been the case for the oldest memories. It is also possible that a victim of bullying consciously hides information or presents it in certain ways, for unknown reasons. The perspective of others, for example those of the parents, teachers, and bystanders could have added valuable information.

There is also controversy regarding whether children should be used for rating teachers’ behavior [26,27]. However, in the field of bullying, it is important to present the perspective of the children who have experienced the victimization in addition to their experiences of the teacher, especially in regard to how the victims are handled when they report their bullying experience. It should also be argued that the power imbalance between the child and teacher could make it even harder for the informants describing their teachers’ behavior as negative.

## 6. Conclusion and Future Research

Our sample is small, but the findings are in many ways consistent. Most of the victims in these long-lasting cases described absence of warmth from the class teacher, which strongly indicates that such comfort from a significant adult is important in a time of great emotional distress. Furthermore, all the victims criticize the class teacher for not offering what we conceptualize as protective control. This demonstrates that these pupils expect their class teacher to protect them from the bullying.

From our study, we cannot say what approach is the best in performing protective control. One obvious goal is to stop the bullying, and effectivity in this regard is, therefore, important. Another significant issue is the impact different forms of intervention may have on the bullies’ emotions and roles in class and the school community. One should consider different forms of intervention both in regard to short-term effects and long-term benefits [28]. Moreover, schools must consider whether the class teachers need some assistance in communicating with those who bully, as this is not always straight forward [29]. Protective control could possibly also be given by the class teacher via special trained personnel.

Examples of good practice from Norway and Ireland concerning bullying prevention are, for example, anti-bullying programs, such as Olweus and Respect from Norway, and the Donegal program from Ireland. In these programs, the role of the teacher in bullying prevention and intervention is emphasized [30].

A bullying case consists of the following: The perspectives of the pupils who bully, the victims, the parents of both the bully and the victim, and the teachers. These perspectives may be greatly different, as bullying cases probably are tense. Further research should address these different perspectives in general, and in particular the role of the teacher and the school, when intervention is concerned.

From this, it would be useful to try different approaches of intervention towards the whole bullying case system and evaluate short-term and longer-term effects on the different parties, using both qualitative and quantitative methods.
